# Development and evaluation of a duplex RT-qPCR assay for the detection and identification of Mayaro and chikungunya viruses

**DOI:** 10.1128/jcm.00420-25

**Published:** 2025-07-03

**Authors:** Konrad M. Wesselmann, Cécile Baronti, Antoine Nougairède, Laurence Thirion, Xavier de Lamballerie, Remi Charrel, Laura Pezzi

**Affiliations:** 1 Unité des Virus Émergents, Aix-Marseille Univ, Università di Corsica, IRD 190, Inserm 1207, IRBA 128791, Marseille, France; 2 Laboratoire Infections Virales Aiguës et Tropicales, AP-HM Hôpitaux Universitaires de Marseille 36900 , Marseille, France; 3 Centre National de Référence des Arbovirus, Inserm-IRBA, Marseille, France; Mayo Clinic Minnesota, Rochester, Minnesota, USA

**Keywords:** *Alphavirus*, *Togaviridae*, arbovirus, chikungunya virus, Mayaro virus, diagnostic, RT-qPCR

## Abstract

Mayaro virus (MAYV) is a mosquito-borne alphavirus that is widespread in the Amazon basin, where it co-circulates with the closely related chikungunya virus (CHIKV). Due to the limited surveillance and technical limitations of diagnostic assays (scarcity of commercial assays, serology cross-reactivity), the true burden of MAYV is uncertain. We designed a new RT-qPCR assay targeting the nsp1 gene for MAYV detection, which can be used in monoplex or duplex format. In the duplex format, the new MAYV assay is combined with a CHIKV assay and a second MAYV assay, both previously published. The lower limit of detection with a 95% positivity rate was determined to be <10 RNA copies/μL in monoplex and duplex formats for both MAYV and CHIKV. Monoplex and duplex assays proved to be linear within the tested range of approximately 10^8^ to 10^2^ RNA copies/μL and showed 100% specificity against a wide panel of arboviruses as well as several other pathogens in clinical samples. The testing of CHIKV-positive sera and MAYV-spiked plasma samples confirmed the suitability of the assays in a clinical setting. These assays offer a reliable tool for detection and differentiation of MAYV and CHIKV in endemic settings.

**IMPORTANCE:**

Molecular diagnostic capabilities for detecting alphaviruses other than chikungunya virus (CHIKV) remain limited. Mayaro virus (MAYV), an emerging mosquito-borne alphavirus, co-circulates with CHIKV in the Americas, making clinical differentiation between the two viruses challenging. To address this, we developed a novel RT-qPCR assay specifically for the detection of MAYV, which can also be used in a duplex format to simultaneously detect and distinguish CHIKV. The assay was thoroughly evaluated in both monoplex and duplex formats and demonstrated high sensitivity and specificity. This new tool is particularly valuable for the detection of MAYV, especially in resource-limited settings, where its duplex format offers efficient and accurate differentiation of acute infections.

## INTRODUCTION

Mayaro virus (MAYV) (genus *Alphavirus*, family *Togaviridae*) is widely distributed in the Amazon basin and primarily transmitted by tree-dwelling *Haemagogus* mosquitoes (1, 2). Natural infections have also been detected in Aedes mosquitoes; however, their role in MAYV transmission and in the potential establishment and maintenance of an urban transmission cycle remains uncertain (3). Nevertheless, Aedes species may significantly contribute to broader MAYV circulation, raising concerns about its potential spread to new areas, including the Caribbean, where the virus was already isolated in 2015 (4). Three genotypes of MAYV have been identified to date (D, N, and L), with genotype D responsible for the majority of cases (2). High seroprevalence rates have been observed in Brazil, Bolivia, and Peru (2, 5), where the closely related CHIKV is also known to circulate (6). The two pathogens share a similar clinical presentation generally characterized by fever, headache, rash, myalgia, and arthralgia (6). The co-circulation of CHIKV and MAYV, combined with a significant cross-reactivity affecting serological tests, makes it difficult to assess the true extent of MAYV circulation in regions where CHIKV may also be encountered (2, 7, 8). The diagnosis of acute infections relies on molecular tools that can differentiate the two pathogens; however, no commercial RT-qPCR assay is available for MAYV, and few in-house molecular assays have been published in the literature (7).

In this study, we describe and evaluate a newly developed RT-qPCR assay for the detection of MAYV. The assay can be used in either monoplex or duplex format, the latter including an additional previously described MAYV RT-qPCR assay to enhance robustness, along with a CHIKV RT-qPCR assay for the simultaneous detection of the two pathogens in areas where they co-circulate.

## MATERIALS AND METHODS

### Design of a monoplex MAYV_nsp1 RT-qPCR assay and development of a MAYVx2-CHIKV duplex assay

MAYV sequences were retrieved from National Center for Biotechnology Information (NCBI) GenBank (https://www.ncbi.nlm.nih.gov/genbank/) using the search term “Mayaro virus” (search performed on 18/10/2024). Of the 258 available sequences, 48 were excluded because they were labeled “unverified” or not referring to Mayaro virus (Table S1). An alignment was performed using MAFFT (https://mafft.cbrc.jp/; algorithm: auto; scoring matrix BLOSUM62), including all remaining MAYV complete (*n* = 80) and partial (*n* = 130) sequences. These 210 sequences were aligned together, with 17 complete CHIKV sequences covering the genetic diversity of the five CHIKV lineages (Eastern/Central/Southern Africa-ECSA 1–3, West African, and Asian) as well as representative sequences of other alphaviruses (O’nyong-nyong, Ross River, Bebaru, Getah, Semliki Forest, Sindbis, and Una viruses) belonging to the same serocomplex as CHIKV and MAYV according to the International Committee on Taxonomy of Viruses classification (Table S2). The aim of this alignment was to identify a target region that (i) was highly conserved across different MAYV genotypes and (ii) contained sufficient sequence divergence between MAYV, CHIKV, and other alphaviruses to enable the design of MAYV-specific primers and probes.

To develop a duplex MAYV-CHIKV assay, a previously published CHIKV RT-qPCR assay (9) was selected to be used in combination with the new monoplex MAYV RT-qPCR assay (here called MAYV_nsp1). A second monoplex MAYV assay targeting the conjunction of 5′UTR and nsp1 (here called MAYV_5′UTR/nsp1) selected among those available in literature was also incorporated into the duplex format to enhance assay robustness by targeting different genomic regions (10). In this duplex assay, called MAYVx2-CHIKV, the probes of both MAYV assays were labeled with VIC dye, whereas that of the CHIKV monoplex assay was labeled with FAM dye. Primers and probe concentrations for CHIKV and MAYV assays were optimized, and the combination providing the best analytical sensitivity was selected. Details of the MAYV_nsp1 RT-qPCR monoplex and MAYVx2-CHIKV duplex RT-qPCR assays are presented in Table 1. The primers and probes of the MAYV_nsp1 and the MAYVx2-CHIKV assays were produced in a lyophilized format in single glass vials as previously described for other assays (11) and were made available on the EVAM online catalog under the references 001K-06161 and 001K-06162.

**TABLE 1:** Primers and probes included in the newly developed MAYV_nsp1 monoplex and MAYVx2-CHIKV duplex assays

	Reference	Primer/probe	5′→3′ sequence	Target	Position^*a*^	Amplicon size (nt)	Concentration^*b*^
Monoplex MAYV_nsp1 RT-qPCR assay	Developed in this study	MAYV-F	CATCAGGYGAAGTCGTTGACAGA	nsp1	396–418	118	400 nM
		MAYV-R	CCTGCATGTCTGATCTGTGTGA		493–514		200 nM
		MAYV-P	VIC-AAGATAGACGACCTGCAGTC-QSY		431–450		80 nM
Duplex MAYVx2-CHIKV RT-qPCR assay	Developed in this study	MAYV-F	CATCAGGYGAAGTCGTTGACAGA	nsp1	396–418	118	400 nM
		MAYV-R	CCTGCATGTCTGATCTGTGTGA		493–514		200 nM
		MAYV-P	VIC-AAGATAGACGACCTGCAGTC-QSY		431–450		80 nM
	Waggoner et al. (10)	MAYV-F	AAGCTCTTCCTCTGCATTGC	5' UTR-nsp1	51–70	109	300 nM
		MAYV-R1	TGCTGGAAACGCTCTCTGTA		141–160		300 nM
		MAYV-R2	TGCTGGAAATGCTCTTTGTA				300 nM
		MAYV-P	VIC-GCCGAGAGCCCGTTTTTAAAATCAC-QSY		116–140		200 nM
	Pastorino et al. (9)	CHIK-F	AAGCTYCGCGTCCTTTACCAAG	E1	10,380–10,401	208	900 nM
		CHIK-R	CCAAATTGTCCYGGTCTTCCT		10,568–10,588		900 nM
		CHIK-P	FAM-CCAATGTCYTCMGCCTGGACACCTTT-QSY		10,479–10,504		200 nM

^*a*^ Positions refer to the CHIKV "Ross strain" (GenBank accession number MG280943) and MAYV "BR/SJRP/LPV01/2015" (GenBank accession number KT818520).

^*b*^ Concentration in the final reaction mix.

### RT-qPCR

Viral supernatants and clinical samples used in this study were extracted with EZ1 Virus Mini Kit v2.0 on EZ1 Advanced XL (both from QIAGEN, Hilden, Germany). All RT-qPCR reactions were performed using SuperScript III Platinum One-Step RT-qPCR Kit (Invitrogen-Thermo Fisher Scientific, Waltham, MA, USA) on a QuantStudio 12K Flex Real-Time PCR System (Thermo Fisher Scientific, Waltham, MA, USA). The final reaction mix had a volume of 25 µL consisting of 5 µL sample RNA, 12.5 µL of 2× Reaction Mix, 0.5 µL of Superscript III RT/Platinum Taq Mix, and primers and probe at the concentrations described in Table 1. The cycling conditions were as follows: 50°C for 15 min; 95°C for 2 min; and 45 cycles of 95°C for 15 s and 60°C for 45 s. Positive and negative controls were included in each RT-qPCR run.

### Limit of detection

The limits of detection for the monoplex MAYV_nsp1 and duplex MAYVx2-CHIKV assays were evaluated using five quantified CHIKV strains (representing the five CHIKV genetic lineages) and one quantified MAYV strain from genotype D (Table 2). Viral RNA was extracted from cell culture supernatant as previously described and serially diluted. Eight decreasing serial dilutions of each strain with concentrations ranging from approximately 10^2^ to 0 RNA copies/µL were tested in eight replicates each. Ct values ≥ 40 were considered negative. The lower limit of detection was defined as the concentration of viral RNA copies achieving a 95% positivity hit rate (LOD95). The LOD95 was calculated with probit regression analysis using IBM SPSS software version 24.

**TABLE 2:** Strains tested to assess the specificity of the monoplex MAYV_nsp1 RT-qPCR and duplex RT-qPCR MAYVx2-CHIKV assays

Genus	Virus species and acronyms		Strain	Viral load (TCID50/mL)	Reference on EVAg or NCPV catalog
*Alphavirus*	Chikungunya virus	CHIKV-West African	UVE/CHIKV/1983/SN/WA 37997	10^6.22^	001V-02448 (EVAg)
		CHIKV-Asian	H20235/STMARTIN/2013	10^5.57^	001N-EVAg1583 (EVAg)
		CHIKV-ECSA1	UVE/CHIKV/UNK/XX/ROSS	10^6.49^	001v-EVA1455 (EVAg)
		CHIKV-ECSA2	UVE/CHIKV/2011/CD/Brazza_MRS1	10^6.57^	001v-EVA960 (EVAg)
		CHIKV-ECSA3	UVE/CHIKV/2006/RE/LR2006_OPY1	10^8.49^	001v-EVA83 (EVAg)
	Mayaro virus	MAYV	UVE/MAYV/1954/TT/TC625	10^8.82^	001v-EVA502 (EVAg)
	Venezuelan equine encephalitis virus	VEEV	UVE/VEEV/UNK/XX/TC83 vaccine	10^9,42^	001v-EVA1459 (EVAg)
	Western equine encephalitis virus	WEEV	UVE/WEEV/UNK/XX/47a	10^8,32^	001v-EVA1479 (EVAg)
	Eastern equine encephalitis virus	EEEV	UVE/EEEV/1999/XX/H178_99	10 ^7,82^	001v-EVA1480 (EVAg)
	Ross River virus	RRV	5281v	10^8,16^	0005281v (NCPV)
	O'nyong-nyong virus	ONNV	UVE/ONNV/UNK/SN/Dakar 234	10^4,22^	001v-EVA1044 (EVAg)
	Semliki Forest virus	SFV	UVE/SFV/UNK/XX/1745	10^4,42^	001V-02468 (EVAg)
	Sindbis virus	SINV	UVE/SINV/UNK/EG/Egypt 339	10^4,32^	001V-02469 (EVAg)
*Flavivirus*	Dengue virus-1	DENV-1	1579/18	10^7,08^	007V-03123 (EVAg)
	Dengue virus-2	DENV-2	UVE/DENV-2/1998/MQ/H_IMTSSA-MART_98-703	10^4,57^	001v-EVA1019 (EVAg)
	Dengue virus-3	DENV-3	UVE/DENV-3/UNK/PH/H87	10^6,42^	001v-EVA242 (EVAg)
	Dengue virus-4	DENV-4	UVE/DENV-4/2012/BR/4829	10^6,07^	001V-03361 (EVAg)
	Japanese encephalitis virus	JEV	UVE/JEV/2009/LA/CNS769	10^5,57^	001V-02217 (EVAg)
	Saint Louis encephalitis virus	SLEV	UVE/SLEV/UNK/US/MSI-7	10^4,82^	001v-EVA128 (EVAg)
	Tick-borne encephalitis virus	TBEV	UVE/TBEV/2013/FR/32.11 WT-PCR	10^8,82^	001V-02352 (EVAg)
	Zika virus	ZIKV	UVE/ZIKV/2013/PF/Papeete 1	10^6,82^	001V-02547 (EVAg)
	Yellow fever virus	YFV	UVE/YFV/UNK/XX/Vaccinal strain 17D	10^4.32^	001v-EVA67 (EVAg)
	West Nile virus	WNV	UVE/WNV/2008/US/R94224	10^7,32^	001V-02224 (EVAg)
	Usutu virus	USUV	UVE/USUV/1959/ZA/SAAR-1776	10^5,32^	001v-EVA138 (EVAg)
	Murray Valley encephalitis virus strain	MVEV	UVE/MVEV/UNK/AU/3329	10^4,32^	001v-EVA145 (EVAg)
*Phlebovirus*	Toscana virus	TOSV	UVE/TOSV/2014/FR/5904	10^7,42^	001V-02461 (EVAg)

### Linear range

The linear range of the MAYV and CHIKV monoplex assays as well as of the duplex MAYVx2-CHIKV assay was assessed using 10-fold serial dilutions of MAYV and CHIKV, ranging approximately from 10^8^ to 10^2^ viral RNA copies/µL. Two replicates of each dilution were tested per run. The linear correlation coefficient (*R*^2^) and qPCR efficiency were calculated for each assay.

### Specificity

The specificity of the MAYV_nsp1 and MAYVx2-CHIKV duplex assays was assessed using a panel of 21 related and unrelated viruses from *Alphavirus* (*n* = 7), *Flavivirus* (*n* = 12), and *Phlebovirus* (*n* = 1) genera. The MAYV_nsp1 monoplex assay was also tested against the five CHIKV strains representative of different CHIKV lineages. All the viral strains included in the specificity panel were provided by the European Virus Archive (EVAg, https://www.european-virus-archive.com/), except Ross River virus, which was supplied by the National Collection of Pathogenic Viruses (NCPV, https://www.pheculturecollections.org.uk/collections/ncpv.aspx). The specificity panel is presented in Table 2.

### Clinical samples

A total of 47 clinical samples collected from CHIKV-confirmed acute cases were kindly provided by the National Reference Center (NRC) for arboviruses for testing using the MAYVx2-CHIKV duplex and CHIKV monoplex assays included in the duplex assay (9). Because MAYV-positive clinical samples were not available for testing, the qualified-negative plasma provided by the French Blood Bank was spiked with a MAYV strain (for specifications, see Table 2, passage 12 on Vero cells) in 10-fold serial dilutions in order to simulate clinical samples with varying viral loads. Four replicates of each dilution were tested in parallel using MAYV_nsp1, MAYVx2-CHIKV duplex assay, and MAYV_5′UTR/nsp1 monoplex assay (10) included in the duplex assay using the same RT-qPCR conditions.

To further confirm the specificity of the MAYVx2-CHIKV duplex assay, we tested 32 sera from febrile patients of the APHM University Hospitals of Marseille, who initially had clinically suspected CHIKV infections that were CHIKV PCR-negative but revealed to be positive by LAMP (Alethia Malaria, Meridian Bioscience) and thick and thin blood smear for *Plasmodium falciparum* (*n* = 9) and positive by PCR for cytomegalovirus (*n* = 13) (CMV-RGENE, Biomérieux) and Epstein Barr virus (*n* = 10) (EBV-RGENE, Biomérieux).

## RESULTS

### Design of monoplex MAYV_nsp1 RT-qPCR and duplex MAYVx2-CHIKV assay

The genomic sequences of MAYV, CHIKV, and other phylogenetically related alphaviruses were aligned to perform an *in silico* analysis of the newly developed MAYV_nsp1 monoplex as well as to evaluate the CHIKV and MAYV_5′UTR/nsp1 monoplex assays selected from the literature for inclusion in the MAYVx2-CHIKV duplex assay (9, 10). Mismatches between this sequence panel and primers and probe sets were examined, with specific attention to the five 3′ terminal nucleotides of the primers since the mismatches in these positions are especially relevant for assay sensitivity. A conserved region of the non-structural protein 1 (nsp1) was selected as the target of the new MAYV monoplex assay. *In silico* analysis highlighted several mismatches between the primers and the probe of the newly developed MAYV_nsp1 monoplex assay and the strains of alphaviruses other than MAYV. Results of the *in silico* analysis are presented in Fig. S1.

The duplex MAYVx2-CHIKV is more precisely a dual-target duplex assay since it combines one monoplex test for CHIKV (9) and two monoplex tests for MAYV: the one developed in-house and a second one already available in literature (10). The inclusion of two assays for MAYV detection provides two key benefits: (i) increased robustness, as previously explained for multi-target assays (12, 13); and (ii) enhanced fluorescent signal intensity compared to a duplex assay with only one monoplex MAYV assay.

### Analytical sensitivity

**Fig 1:**
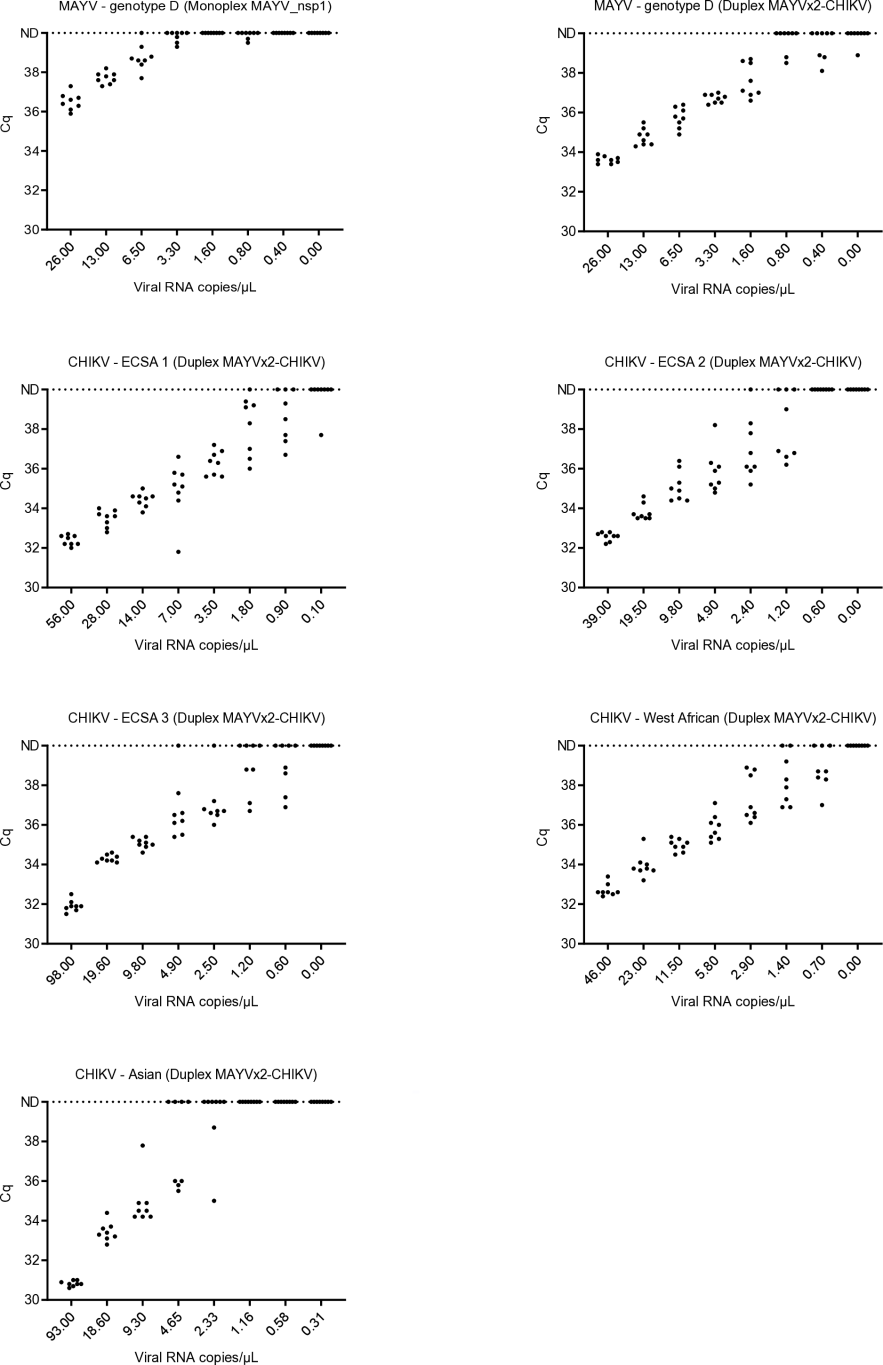
Detection of dilutions of one MAYV strain (genotype D) and five CHIKV strains with the MAYVx2-CHIKV duplex and MAYV_nsp1 monoplex assays developed in this study. ND: not detected.

The LOD95 of the MAYV-nsp1 assay was 7.6 viral RNA copies/µL (confidence interval [CI]: 5.8–12.4). For the duplex MAYVx2-CHIKV assay, LOD95 was 1.7 viral RNA copies/µL for the MAYV strain (CI: 1.2–3.6), 2.0 viral RNA copies/µL for the CHIKV strain—ECSA 1 lineage (CI: 1.5–4.5), 2.6 viral RNA copies/µL for the CHIKV strain—ECSA 2 lineage (CI: 1.8–8.4), 4.7 viral RNA copies/µL for the CHIKV strain—ECSA 3 lineage (CI: 3.3–10.1), 1.8 viral RNA copies/µL for the CHIKV strain—West African lineage (CI: 1.3–3.9), and 7.0 viral RNA copies/µL for the CHIKV strain—Asian lineage (CI: 6.0–13.9) (Table 3; Fig. 1).

**TABLE 3:** Analytical sensitivity of the monoplex MAYV_nsp1 RT-qPCR and duplex RT-qPCR MAYVx2-CHIKV assays

		Monoplex MAYV_nsp1 RT-qPCR assay				Duplex MAYVx2-CHIKV RT-qPCR assay			
	Viral RNA copies/µL	No. of total samples tested	No. of positive samples	Ct mean	LOD95 [CI]	No. of total samples tested	No. of positive samples	Ct mean	LOD95 [CI]
Mayaro virus (strain UVE/MAYV/1954/TT/TC625)	26	8	8/8	36.5	7.6 viral RNA copies/µL [5.8–12.4]	8	8/8	33.6	1.7 viral RNA copies/µL [1.2–3.6]
	13	8	8/8	37.7		8	8/8	34.8	
	6.5	8	7/8	38.8		8	8/8	35.7	
	3.3	8	3/8	39.5		8	8/8	36.7	
	1.6	8	0/8	ND		8	8/8	37.6	
	0.8	8	2/8	39.6		8	2/8	38.7	
	0.4	8	0/8	ND		8	3/8	38.6	
	0	8	0/8	ND		8	1/8	38.9	
Chikungunya virus (strain UVE/CHIKV/UNK/XX/ROSS)—ECSA 1 lineage	56	No amplification				8	8/8	32.4	2.0 viral RNA copies/µL [1.5–4.5]
	28					8	8/8	33.5	
	14					8	8/8	34.4	
	7					8	8/8	34.9	
	3.5					8	8/8	36.3	
	1.8					8	7/8	37.9	
	0.9					8	5/8	37.9	
	0					8	1/8	37.7	
Chikungunya virus (strain UVE/CHIKV/2011/CD/Brazza_MRS1)—ECSA 2 lineage	39	No amplification				8	8/8	32.6	2.6 viral RNA copies/µL [1.8–8.4]
	19.5					8	8/8	33.8	
	9.8					8	8/8	35.1	
	4.9					8	8/8	35.9	
	2.4					8	7/8	36.6	
	1.2					8	5/8	37.1	
	0.6					8	0/8	ND	
	0					8	0/8	ND	
Chikungunya virus (strain UVE/CHIKV/2006/RE/LR2006_OPY1)—ECSA 3 lineage	98	No amplification				8	8/8	31.9	4.7 viral RNA copies/µL [3.3–10.1]
	19.6					8	8/8	34.3	
	9.8					8	8/8	35.1	
	4.9					8	7/8	36.3	
	2.5					8	7/8	36.6	
	1.2					8	4/8	37.9	
	0.6					8	4/8	38.0	
	0					8	0/8	ND	
Chikungunya virus (strain UVE/CHIKV/1983/SN/WA 37997)—West African lineage	46	No amplification				8	8/8	32.7	1.8 viral RNA copies/µL [1.3–3.9]
	23					8	8/8	34.0	
	11.5					8	8/8	35.0	
	5.8					8	8/8	35.9	
	2.9					8	8/8	37.3	
	1.4					8	6/8	37.8	
	0.7					8	5/8	38.2	
	0					8	0/8	ND	
Chikungunya virus (strain H20235/STMARTIN/2013)—Asian lineage	93	No amplification				8	8/8	30.8	7.0 viral RNA copies/µL [6.0–13.9]
	18.6					8	8/8	33.4	
	9.3					8	8/8	34.9	
	4.7					8	4/8	35.8	
	2.3					8	2/8	36.9	
	1.2					8	0/8	ND	
	0.6					8	0/8	ND	
	0					8	0/8	ND	

### Linearity

**Fig 2:**
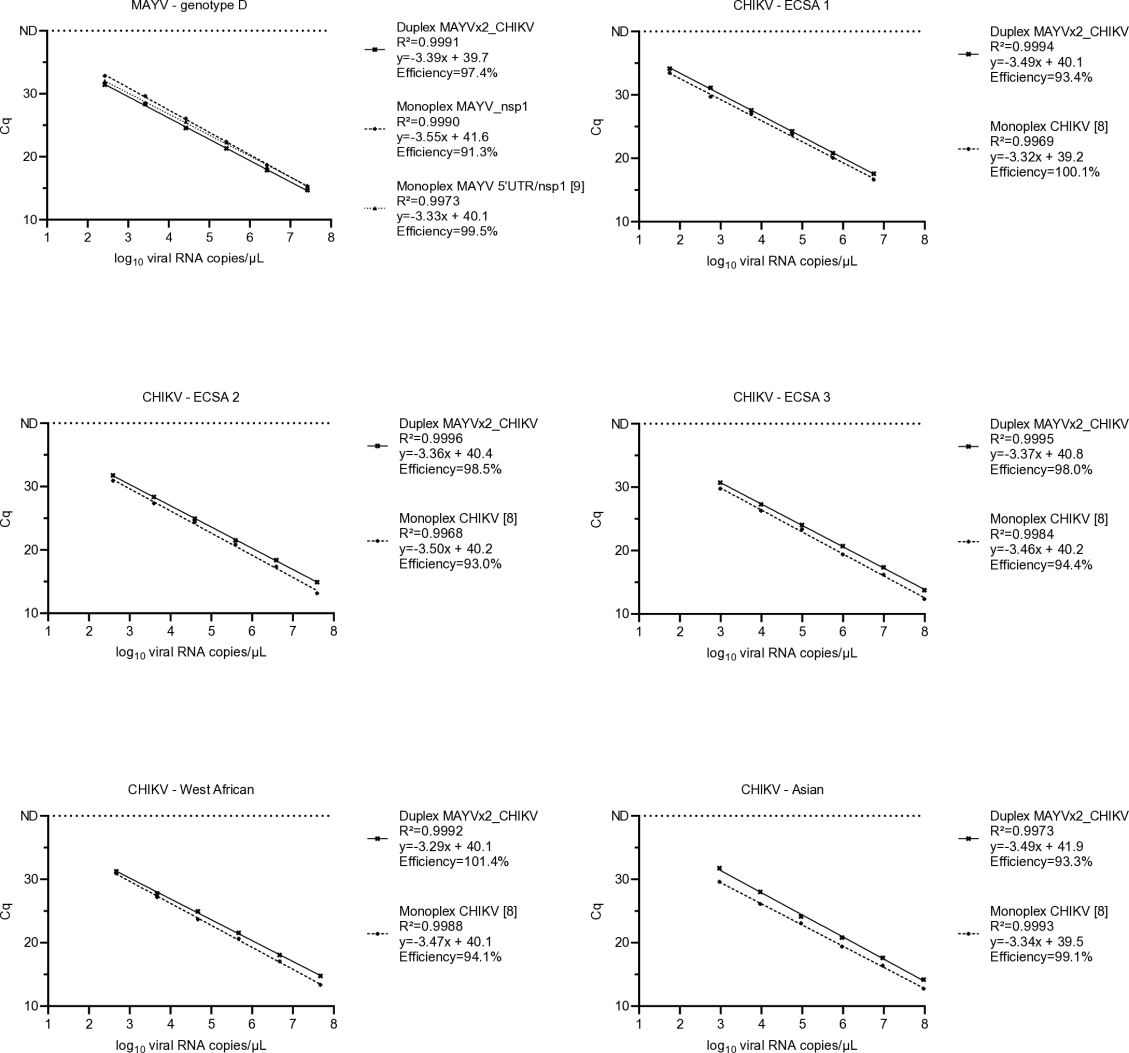
Linearity of the duplex MAYVx2-CHIKV and monoplex assays included in the duplex assay. Each dilution was tested in duplicate in a single run, and mean Cq is represented. ND: not detected.

The MAYVx2-CHIKV and the three monoplex assays were linear within the tested concentrations ranging from 10^8^ to 10^2^ viral RNA copies/μL of MAYV and CHIKV strains, with *R*^2^ > 0.99 and qPCR efficiencies > 91% (Fig. 2). The assays demonstrated a consistent efficiency of amplification within the desired range of 90–100% (14).

### Specificity

No amplification was observed when using both the MAYV_nsp1 and duplex MAYVx2-CHIKV assays on the specificity panel containing other arboviruses (for details, see Table 2). Thus, the MAYV_nsp1 assay demonstrated 100% specificity for MAYV, while the MAYVx2-CHIKV duplex assay proved to be 100% specific for CHIKV (in FAM channel) and MAYV (in VIC channel).

### Clinical samples

Among the 47 clinical samples from CHIKV-confirmed cases, 43 were positive when tested with the monoplex CHIKV assay (9). The MAYVx2-CHIKV duplex assay detected the same 43 samples and an additional three weakly positive samples (Ct value > 36). The ΔCq between the MAYVx2-CHIKV duplex and CHIKV monoplex assays was generally low (<1 Cq), except for one weakly positive sample (Cq > 35, ΔCq = 2.5). The results of the molecular testing on CHIKV-positive clinical samples are presented in Table S3.

As no MAYV-positive clinical samples were available, we used MAYV-spiked plasma covering a wide range of MAYV concentrations. All samples tested positive by all three tested assays (MAYVx2-CHIKV duplex assay and the two MAYV monoplex assays included in the MAYVx2-CHIKV duplex), except for the least concentrated sample, which was not detected by the MAYVx2-CHIKV duplex assay. For the other samples, the MAYVx2-CHIKV duplex assay presented lower Cq values than the monoplex assays (ΔCq = 1.4–3.5). Notably, fluorescence signals were highest in the MAYVx2-CHIKV duplex and lowest in the MAYV_5′UTR/nsp1 assay (10) (data not shown). Results are presented in Table S4.

Additionally, both the MAYVx2-CHIKV duplex and MAYV_nsp1 assays tested negative in all 32 sera from patients suspected of CHIKV but infected with other pathogens. This confirmed the absence of cross-reactivity with these etiological agents and the matrices used (plasma and whole blood).

## DISCUSSION

MAYV and CHIKV co-circulate in extensive regions of South America, as documented by several studies. MAYV circulated at low levels in urban areas of Trinidad and Tobago during the CHIKV explosive outbreak in 2014 (15); more recently, CHIKV emerged in a Brazilian region of the Amazon Forest that is endemic for MAYV (16). CHIKV and MAYV co-infections have also been reported (17). Despite these pieces of evidence, while CHIKV is routinely included in the differential diagnosis of febrile presentation, MAYV is not. A recent external quality assessment (EQA) by EVD-LabNet (https://www.ecdc.europa.eu/en/about-us/partnerships-and-networks/disease-and-laboratory-networks/evd-labnet) revealed that while most European reference laboratories could reliably detect CHIKV, diagnostic capacity for MAYV was inadequate, with nearly 40% of laboratories lacking a MAYV-specific molecular test (18). Since MAYV is commonly associated with travelers visiting the Amazonian forest, this scarce diagnostic capability could result in imported MAYV cases going undiagnosed.

We developed here a sensitive and specific MAYV RT-qPCR; its use in a duplex format, combined with a CHIKV assay, can facilitate the inclusion of MAYV in the differential diagnosis panel, optimizing time, resources, and sample volume at equal cost. Furthermore, incorporating a second MAYV assay in the duplex MAYVx2-CHIKV assay enhances the test robustness. As previously emphasized (12, 13), the advantage of targeting multiple genomic regions is to minimize the risk of false-negative results due to mutations, which are often observed in emerging pathogens, particularly RNA viruses, due to higher evolutionary rates than DNA pathogens (19).

This study has some limitations. MAYV-positive clinical samples were unavailable for testing; however, based on our experience, matrices spiked with viral strains serve as a reliable surrogate for clinical samples (20, 21). Yet, the MAYV strain used in this study is an ancestral one (isolated in 1954). Monoplex and duplex assays will need to be validated with more recent isolates. Furthermore, the design of our assay is limited by the small number of available MAYV genomes. Currently, only three genotypes are known, with very limited genetic data for the recently identified genotype D. Additionally, the existence of other, yet undetected, genotypes remains possible. As more MAYV sequence data become available in the coming years, a re-evaluation of our MAYV RT-qPCR assay will be necessary to ensure it captures the full genomic diversity of MAYV. In the meantime, *in silico* analysis demonstrated the assay reliability in detecting all MAYV genotypes, with no mismatches identified, a finding confirmed by *in vitro* analysis using a genotype D strain. Moreover, as previously explained, the combination of two MAYV assays (nsp1 together with 5′UTR/nsp1 [10]) targeting a highly conserved genomic region further reduces the risk of false-negative results, particularly in the event of the emergence of a new MAYV genotype. Lastly, this evaluation merits being complemented through testing clinical specimens from an endemic area.

Although certain aspects of acute MAYV infections remain to be fully understood (such as the duration of the viremic window, viremia levels, and the most useful biological fluids for RT-qPCR), the new molecular test for MAYV detection allows physicians to more easily screen for MAYV infection in cases of febrile presentations with arthralgia. Furthermore, researchers can leverage this tool in entomological and epidemiological investigations. This will increase awareness about MAYV and allow for an increase in the knowledge of MAYV in humans, in the animal reservoir, and in arthropod vectors.

## SUPPLEMENTAL MATERIAL

Supplemental figures and tables jcm.00420-25-s0001.docx Fig. S1 to S3 and Table S1 to S4.

ASM does not own the copyrights to Supplemental Material that may be linked to, or accessed through, an article. The authors have granted ASM a non-exclusive, world-wide license to publish the Supplemental Material files. Please contact the corresponding author directly for reuse.
