# Supplementary material for: Development and evaluation of a duplex RT-qPCR assay for the detection and identification of Mayaro and chikungunya viruses
Source: J Clin Microbiol. 2025 Jul 3;63(8):e00420-25. doi: 10.1128/jcm.00420-25 (PMC12345196; doi:10.1128/jcm.00420-25)
Supplement: Supplemental figures and tables — Fig. S1 to S3 and Table S1 to S4. [file jcm.00420-25-s0001.docx]

Supplementary Materials

**Development and evaluation of a duplex RT-qPCR assay for the detection and identification of Mayaro and chikungunya virus**

Konrad M. Wesselmann^a*^, Cécile Baronti^a^, Antoine Nougairède^a,b^, Laurence Thirion^a^, Xavier de Lamballerie^a,b,c^, Remi Charrel^a,b#^, Laura Pezzi^a,c#^

^a^Unité des Virus Émergents (UVE: Aix-Marseille Univ, Università di Corsica, IRD 190, Inserm 1207, IRBA), France

^b^APHM Hôpitaux Universitaires de Marseille, Laboratoire « Infections Virales Aiguës et Tropicales », Marseille, France

^c^Centre National de Référence des Arbovirus, Inserm-IRBA, Marseille, France

#, equal contribution

*Address correspondence to Konrad M Wesselmann: [konrad.wesselmann@univ-amu.fr](mailto:konrad.wesselmann@univ-amu.fr)

A)


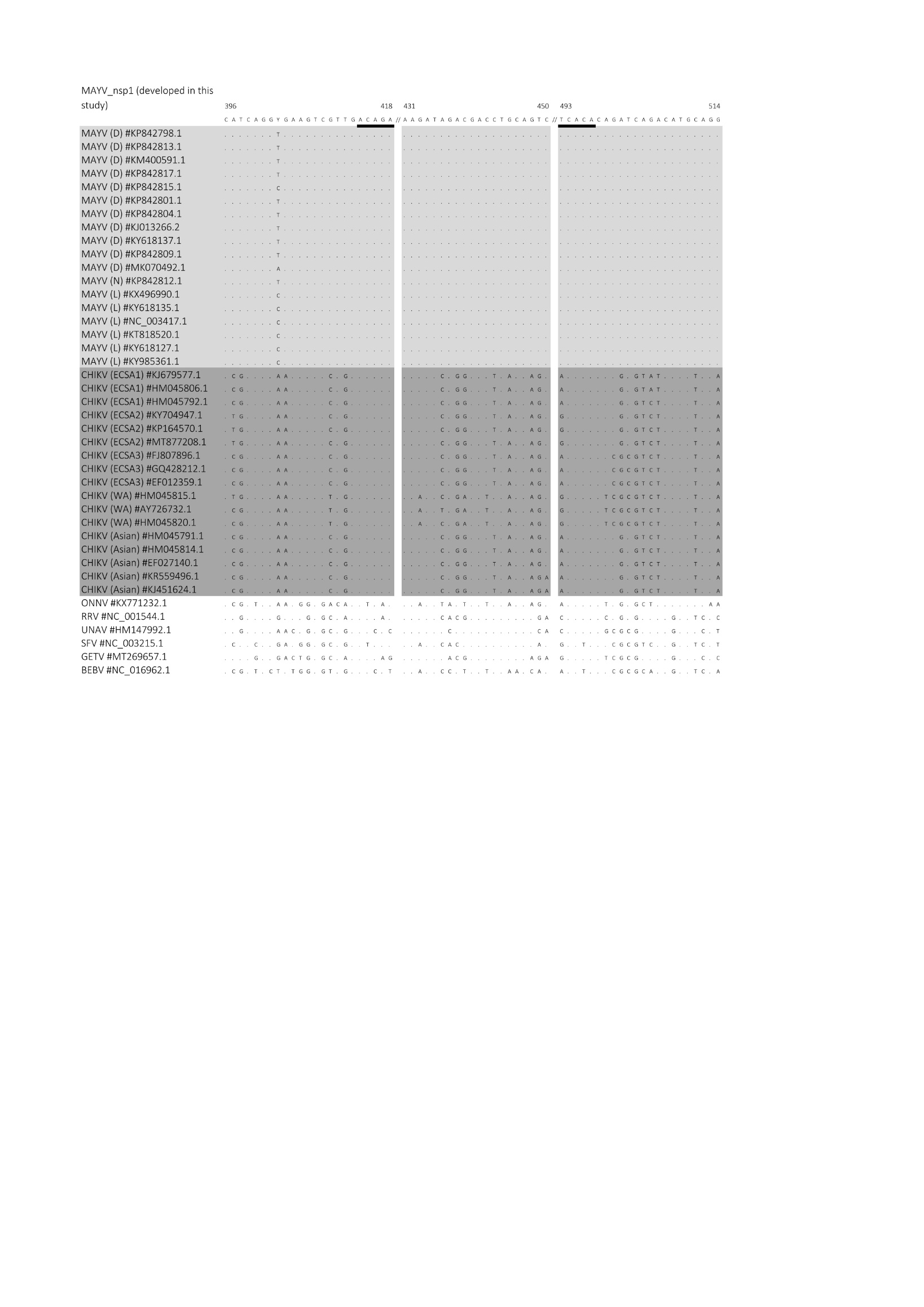


B)


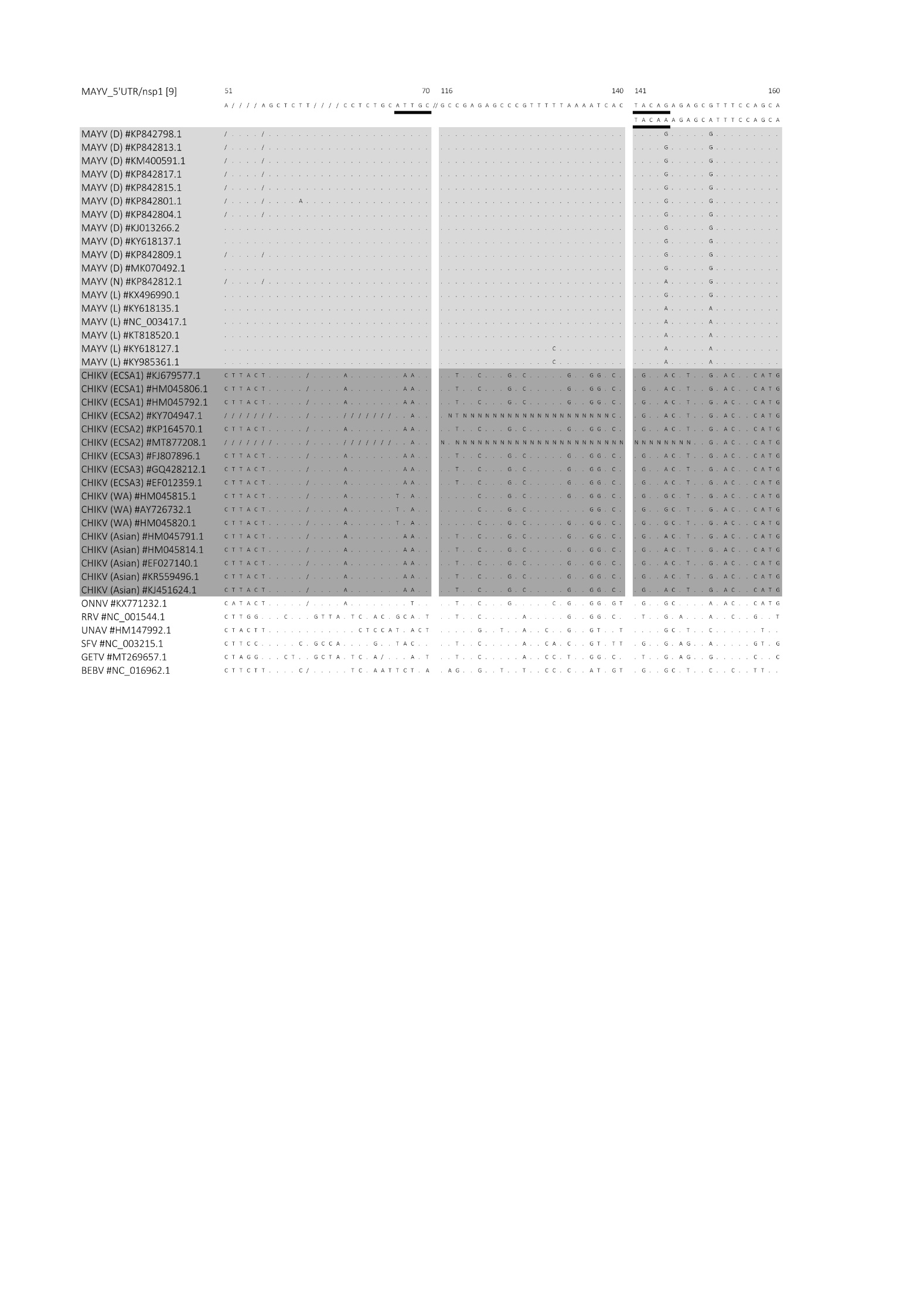


C)


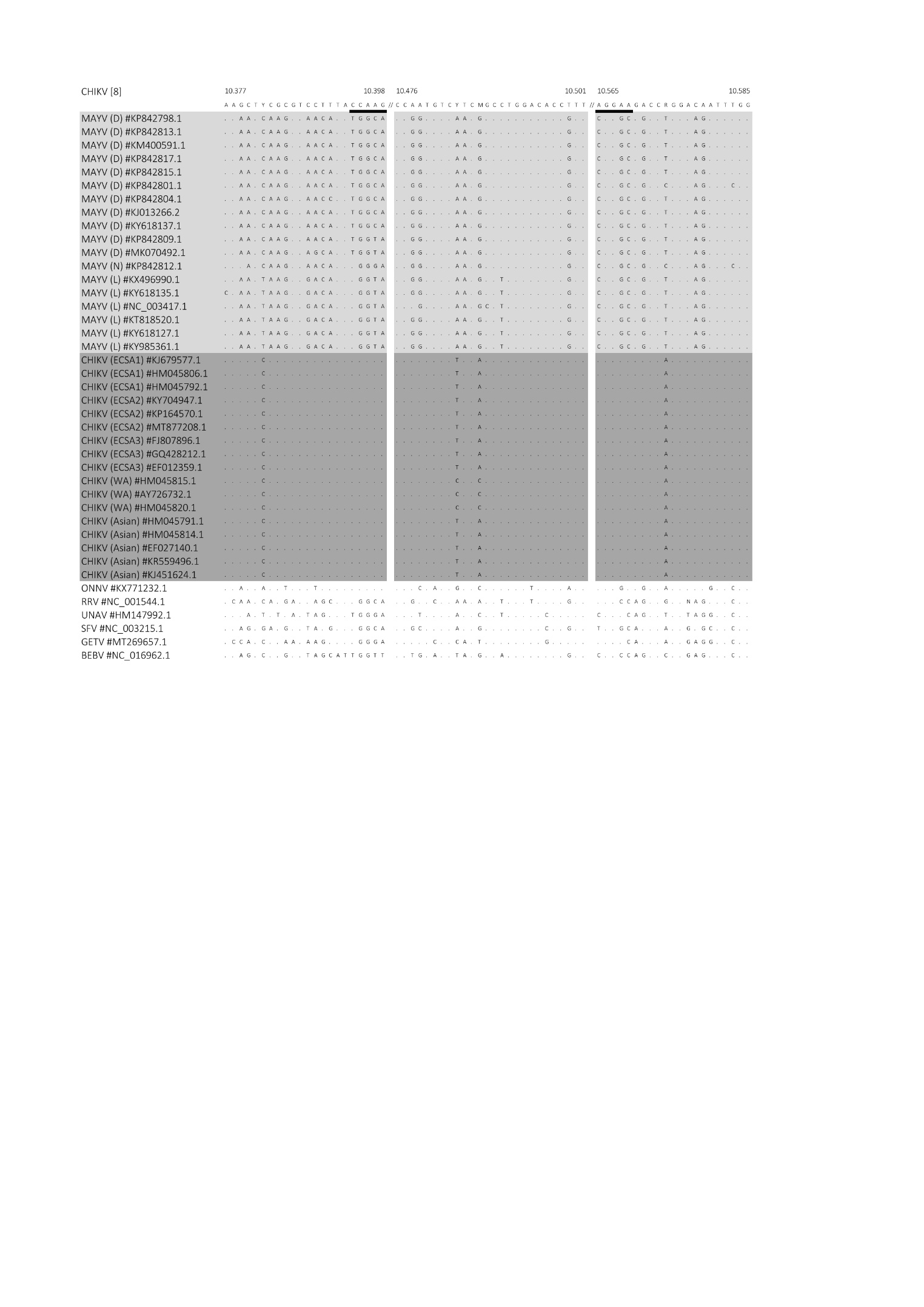


Figure S1. *In silico* analysis of the newly developed MAYV_nsp1 monoplex assay (Figure 1A), the second MAYV monoplex assay (MAYV_5’UTR/nsp1, Figure 1B) and CHIKV monoplex assay included in the MAYVx2-CHIKV duplex assay (Figure 1C). In dark grey, selection of representative MAYV strains; in light grey, CHIKV virus strains; in white, other closely related Alphaviruses. GenBank Accession number of nucleotide position reference sequences: KT818520 for S1A and S1B; MG280943 for S1C. The five 3’ nucleotides of primers are underlined because mismatches in these positions are of particular relevance for assay sensitivity.

Table S1: Accession numbers of excluded sequences and reason of exclusion.

| **GenBank Accession N°** | **Reason of exclusion** |
| --- | --- |
| #PP963513.1 | Labelled “UNVERIFIED” |
| #ON601001.1 | Labelled “UNVERIFIED” |
| #ON601000.1 | Labelled “UNVERIFIED” |
| #OR726664.1 | Labelled “UNVERIFIED” |
| #OR726663.1 | Labelled “UNVERIFIED” |
| #OR726662.1 | Labelled “UNVERIFIED” |
| #OR726661.1 | Labelled “UNVERIFIED” |
| #OR726660.1 | Labelled “UNVERIFIED” |
| #MT247712.1 | Labelled “UNVERIFIED” |
| #MT247711.1 | Labelled “UNVERIFIED” |
| #MH330401.1 | Labelled “UNVERIFIED” |
| #KF770748.1 | Labelled “UNVERIFIED” |
| #OF092884.1 | Not referring to MAYV genomic RNA |
| #LY683327.1 | Not referring to MAYV genomic RNA |
| #GN354399.1 | Not referring to MAYV genomic RNA |
| #GN354398.1 | Not referring to MAYV genomic RNA |
| #GN354397.1 | Not referring to MAYV genomic RNA |
| #GN354396.1 | Not referring to MAYV genomic RNA |
| #GN354395.1 | Not referring to MAYV genomic RNA |
| #GN354394.1 | Not referring to MAYV genomic RNA |
| #GN354393.1 | Not referring to MAYV genomic RNA |
| #GN354392.1 | Not referring to MAYV genomic RNA |
| #GN354391.1 | Not referring to MAYV genomic RNA |
| #GN354390.1 | Not referring to MAYV genomic RNA |
| #NM_001348353.1 | Not referring to MAYV genomic RNA |
| #NM_002095.6 | Not referring to MAYV genomic RNA |
| #NM_145659.3 | Not referring to MAYV genomic RNA |
| #JACVVK020000034.1 | Not referring to MAYV genomic RNA |
| #JAHWGI010000979.1 | Not referring to MAYV genomic RNA |
| #CM033486.1 | Not referring to MAYV genomic RNA |
| #JAHUZN010000003.1 | Not referring to MAYV genomic RNA |
| #MH252920.1 | Not referring to MAYV genomic RNA |
| #MH252919.1 | Not referring to MAYV genomic RNA |
| #MH252918.1 | Not referring to MAYV genomic RNA |
| #MH252915.1 | Not referring to MAYV genomic RNA |
| #MH252914.1 | Not referring to MAYV genomic RNA |
| #MH027651.1 | Not referring to MAYV genomic RNA |
| #MH396439.1 | Not referring to MAYV genomic RNA |
| #KF305671.1 | Not referring to MAYV genomic RNA |
| #KF305670.1 | Not referring to MAYV genomic RNA |
| #LF621737.1 | Not referring to MAYV genomic RNA |
| #KP742343.1 | Not referring to MAYV genomic RNA |
| #KP710881.1 | Not referring to MAYV genomic RNA |
| #KP710880.1 | Not referring to MAYV genomic RNA |
| #KP710879.1 | Not referring to MAYV genomic RNA |
| #LG018151.1 | Not referring to MAYV genomic RNA |
| #HW822105.1 | Not referring to MAYV genomic RNA |
| #DI450490.1 | Not referring to MAYV genomic RNA |

Table S2: Accession numbers of sequences used for the design of MAYV_nsp1 RT-qPCR assay

| **CHIKV** | **MAYV** | | | | **Other alphaviruses** |
| --- | --- | --- | --- | --- | --- |
| Asian #EF027140.1 | #AF023285.1 | #KF305672.1 | #KX496990.1 | #PP505831.1 | BEBV #AF398376.1 |
| Asian #HM045791.1 | #AF126873.1 | #KJ013266.2 | #KY026195.1 | #PP505832.1 | GETV #MT269657.1 |
| Asian #HM045814.1 | #AF126874.1 | #KJ682316.1 | #KY026196.1 | #U94602.1 | ONNV# |
| Asian #KJ451624.1 | #AF126875.1 | #KJ713282.1 | #KY026197.1 |  | RRV #MN038285.1 |
| Asian #KR559496.1 | #AF237947.1 | #KJ739869.1 | #KY026198.1 |  | SFV #AF192909.1 |
| ECSA 1 #HM045806.1 | #AF339482.1 | #KJ742385.1 | #KY026199.1 |  | SINV #NC_001547.1 |
| ECSA 1 #HM045792.1 | #AF398378.1 | #KJ870987.1 | #KY026200.1 |  | UNAV #AF398381.1 |
| ECSA 1 #KJ679577.1 | #AY348561.1 | #KJ879253.1 | #KY618127.1 |  |  |
| ECSA 2 #MT877208.1 | #DQ001069.1 | #KJ879254.1 | #KY618128.1 |  |  |
| ECSA 2 #KP164570.1 | #DQ138315.1 | #KJ879255.1 | #KY618129.1 |  |  |
| ECSA 2 #KY704947.1 | #DQ138316.1 | #KJ879256.1 | #KY618130.1 |  |  |
| ECSA 3 #EF012359.1 | #DQ138317.1 | #KJ879257.1 | #KY618131.1 |  |  |
| ECSA 3 #FJ807896.1 | #DQ138318.1 | #KJ879258.1 | #KY618132.1 |  |  |
| ECSA 3 #GQ428212.1 | #DQ138319.1 | #KJ879259.1 | #KY618133.1 |  |  |
| WA #AY726732.1 | #DQ138320.1 | #KJ879333.1 | #KY618134.1 |  |  |
| WA #HM045815.1 | #DQ487369.1 | #KM400591.1 | #KY618135.1 |  |  |
| WA #HM045820.1 | #DQ487370.1 | #KM400592.1 | #KY618136.1 |  |  |
|  | #DQ487378.1 | #KM400593.1 | #KY618137.1 |  |  |
|  | #DQ487379.1 | #KM400594.1 | #KY618138.1 |  |  |
|  | #DQ487380.1 | #KM400595.1 | #KY618139.1 |  |  |
|  | #DQ487381.1 | #KM400596.1 | #KY618140.1 |  |  |
|  | #DQ487382.1 | #KM400597.1 | #KY985361.1 |  |  |
|  | #DQ487383.1 | #KM400598.1 | #MH252916.1 |  |  |
|  | #DQ487384.1 | #KM400599.1 | #MH252917.1 |  |  |
|  | #DQ487385.1 | #KM400600.1 | #MH330400.1 |  |  |
|  | #DQ487386.1 | #KP710882.1 | #MH330402.1 |  |  |
|  | #DQ487387.1 | #KP710883.1 | #MH330403.1 |  |  |
|  | #DQ487388.1 | #KP710884.1 | #MH513597.1 |  |  |
|  | #DQ487389.1 | #KP710885.1 | #MH938336.1 |  |  |
|  | #DQ487390.1 | #KP710886.1 | #MH938337.1 |  |  |
|  | #DQ487391.1 | #KP710887.1 | #MK070491.1 |  |  |
|  | #DQ487392.1 | #KP710888.1 | #MK070492.1 |  |  |
|  | #DQ487393.1 | #KP710889.1 | #MK288026.1 |  |  |
|  | #DQ487394.1 | #KP710890.1 | #MK573238.1 |  |  |
|  | #DQ487395.1 | #KP710891.1 | #MK573239.1 |  |  |
|  | #DQ487396.1 | #KP710892.1 | #MK573240.1 |  |  |
|  | #DQ487397.1 | #KP710893.1 | #MK573241.1 |  |  |
|  | #DQ487398.1 | #KP742341.1 | #MK573242.1 |  |  |
|  | #DQ487399.1 | #KP742342.1 | #MK573243.1 |  |  |
|  | #DQ487400.1 | #KP842794.1 | #MK573244.1 |  |  |
|  | #DQ487401.1 | #KP842795.1 | #MK573245.1 |  |  |
|  | #DQ487402.1 | #KP842796.1 | #MK573246.1 |  |  |
|  | #DQ487403.1 | #KP842797.1 | #MK837006.1 |  |  |
|  | #DQ487404.1 | #KP842798.1 | #MK837007.1 |  |  |
|  | #DQ487405.1 | #KP842799.1 | #MK956954.1 |  |  |
|  | #DQ487406.1 | #KP842800.1 | #MN138459.1 |  |  |
|  | #DQ487407.1 | #KP842801.1 | #MT227562.1 |  |  |
|  | #DQ487408.1 | #KP842802.1 | #MZ343555.1 |  |  |
|  | #DQ487409.1 | #KP842803.1 | #MZ343556.1 |  |  |
|  | #DQ487410.1 | #KP842804.1 | #MZ343557.1 |  |  |
|  | #DQ487413.1 | #KP842805.1 | #MZ343558.1 |  |  |
|  | #DQ487414.1 | #KP842806.1 | #MZ343559.1 |  |  |
|  | #DQ487415.1 | #KP842807.1 | #MZ343560.1 |  |  |
|  | #DQ487416.1 | #KP842808.1 | #MZ343561.1 |  |  |
|  | #DQ487418.1 | #KP842809.1 | #MZ343562.1 |  |  |
|  | #DQ487419.1 | #KP842810.1 | #MZ962428.1 |  |  |
|  | #DQ487420.1 | #KP842811.1 | #MZ962429.1 |  |  |
|  | #DQ487421.1 | #KP842812.1 | #MZ962430.1 |  |  |
|  | #DQ487422.1 | #KP842813.1 | #NC_003417.1 |  |  |
|  | #DQ487423.1 | #KP842814.1 | #OM718766.1 |  |  |
|  | #DQ487424.1 | #KP842815.1 | #OM718767.1 |  |  |
|  | #DQ487425.1 | #KP842816.1 | #OM718768.1 |  |  |
|  | #DQ487426.1 | #KP842817.1 | #OM718769.1 |  |  |
|  | #DQ487427.1 | #KP842818.1 | #OM718770.1 |  |  |
|  | #DQ487428.1 | #KP842819.1 | #OM718771.1 |  |  |
|  | #DQ487429.1 | #KP842820.1 | #OP628214.1 |  |  |
|  | #DQ487430.1 | #KP954632.1 | #PP339762.1 |  |  |
|  | #DQ487431.1 | #KT754168.1 | #PP339763.1 |  |  |
|  | #HQ664947.2 | #KT818520.1 | #PP339764.1 |  |  |

Table S3: CHIKV-positive clinical samples, tested with CHIKV monoplex assay (included in the MAYVx2-CHIKV duplex assay) and MAYVx2-CHIKV duplex assay. Samples #1 to #7 have been diluted to simulate weakly positive samples.

| **Sample ID** | **Cq value (MAYVx2-CHIKV duplex)** | **Cq value (CHIKV monoplex assay)** | **ΔCq value (Cq value MAYVx2-CHIKV duplex Cq CHIKV monoplex assay)** |
| --- | --- | --- | --- |
| #1 | 37.7 | 35.2 | 2.5 |
| #2 | 37.0 | ND | - |
| #3 | ND | ND | - |
| #4 | 34.6 | 33.8 | 0.8 |
| #5 | 36.2 | ND | - |
| #6 | 36.3 | ND | - |
| #7 | 35.7 | 35.0 | 0.7 |
| #8 | 22.0 | 21.8 | 0.2 |
| #9 | 24.0 | 23.5 | 0.5 |
| #10 | 24.0 | 23.4 | 0.6 |
| #11 | 22.1 | 21.9 | 0.2 |
| #12 | 23.7 | 23.5 | 0.2 |
| #13 | 22.0 | 21.8 | 0.2 |
| #14 | 24.0 | 23.5 | 0.5 |
| #15 | 23.7 | 23.2 | 0.5 |
| #16 | 24.3 | 24.0 | 0.3 |
| #17 | 20.5 | 20.2 | 0.3 |
| #18 | 23.5 | 23.1 | 0.4 |
| #19 | 21.4 | 21.1 | 0.3 |
| #20 | 23.7 | 23.3 | 0.4 |
| #21 | 20.2 | 19.9 | 0.3 |
| #22 | 24.8 | 24.6 | 0.4 |
| #23 | 21.8 | 21.5 | 0.3 |
| #24 | 22.1 | 21.2 | 0.9 |
| #25 | 23.5 | 23.0 | 0.5 |
| #26 | 24.6 | 24.2 | 0.4 |
| #27 | 22.9 | 22.7 | 0.2 |
| #28 | 21.7 | 21.6 | 0.1 |
| #29 | 17.7 | 17.4 | 0.3 |
| #30 | 20.5 | 20.4 | 0.1 |
| #31 | 20.5 | 20.2 | 0.3 |
| #32 | 18.1 | 18.0 | 0.1 |
| #33 | 17.2 | 16.8 | 0.4 |
| #34 | 20.1 | 19.8 | 0.3 |
| #35 | 19.1 | 18.9 | 0.2 |
| #36 | 17.0 | 16.8 | 0.2 |
| #37 | 17.6 | 17.7 | -0.1 |
| #38 | 19.1 | 19.0 | 0.1 |
| #39 | 19.6 | 19.3 | 0.3 |
| #40 | 19.8 | 19.4 | 0.4 |
| #41 | 18.9 | 18.7 | 0.2 |
| #42 | 17.3 | 16.9 | 0.4 |
| #43 | 18.9 | 18.6 | 0.3 |
| #44 | 16.9 | 16.8 | 0.1 |
| #45 | 19.2 | 18.8 | 0.4 |
| #46 | 18.3 | 18.1 | 0.2 |
| #47 | 19.7 | 19.2 | 0.5 |

Table S4: MAYV-spiked plasma samples tested with MAYV_nsp1 monoplex assays and MAYVx2-CHIKV duplex assay.

| **Dilution** | **Average Cq value MAYVx2-CHIKV duplex (SD)** | **Average Cq value MAYV_nsp1 monoplex assay developed in this study (SD)** | **ΔCq value (Cq value MAYVx2-CHIKV duplex – Cq value MAYV_nsp1 monoplex assay developed in this study)** | **Average Cq value MAYV monoplex [9] (SD)** | **ΔCq value (Cq value MAYVx2-CHIKV duplex – Cq value MAYV monoplex assay [9])** |
| --- | --- | --- | --- | --- | --- |
| E-1 | 17.1 (0.5) | 19.3 (0.5) | -2.2 | 20.3 (0.1) | -3.2 |
| E-2 | 20.8 (0.1) | 22.9 (0.0) | -2.1 | 24.3 (0.0) | -3.5 |
| E-3 | 24.1 (0.1) | 25.9 (0.1) | -1.8 | 27.6 (0.0) | -3.5 |
| E-4 | 27.5 (0.2) | 29.0 (0.1) | -1.5 | 30.7 (0.0) | -1.5 |
| E-5 | 30.9 (0.1) | 32.6 (0.2) | -1.7 | 34.2 (0.0) | -3.3 |
| E-6 | 35.3 (1.0) | 36.7 (0.3) | -1.4 | 38.1 (0.1) | -2.8 |
| E-7 | ND | 38.5 (0.7) | NA | 39.2 (0.2) | NA |


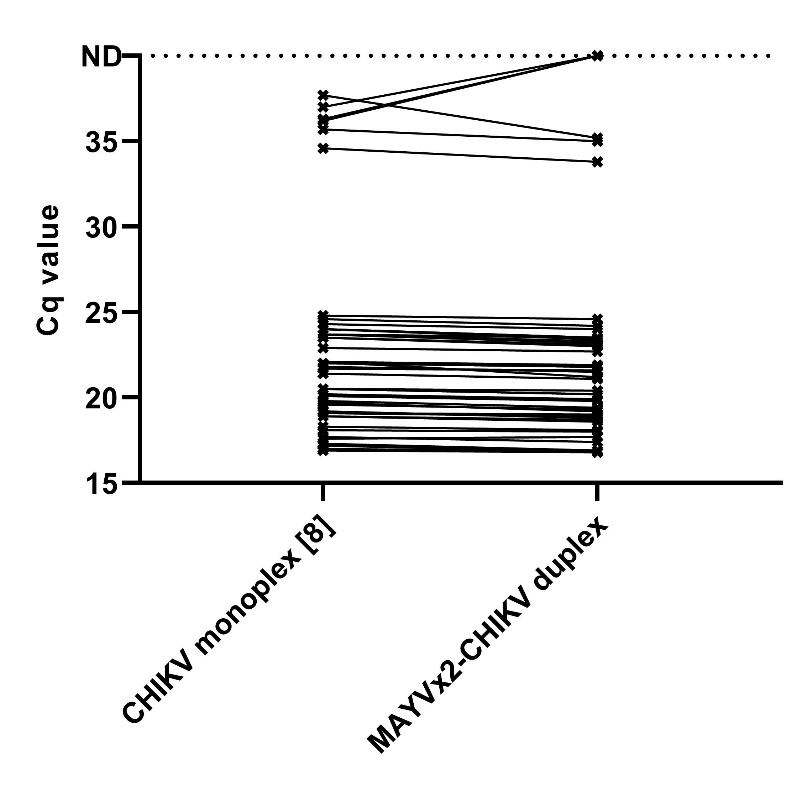


Figure S2: CHIKV-positive clinical samples, tested with CHIKV monoplex assay (included in the MAYVx2-CHIKV duplex assay) [8] and MAYVx2-CHIKV duplex assay.


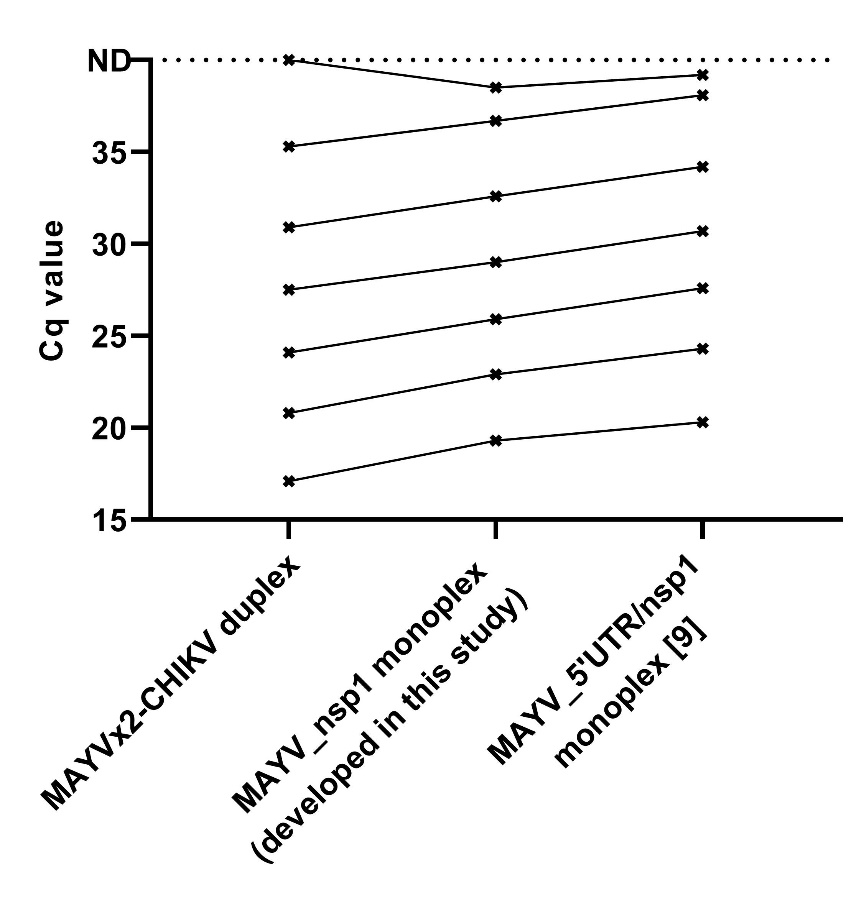


Figure S3: MAYV-spiked plasma samples tested with MAYV monoplex assays (MAYV_nsp1 developed in this study and MAYV_5’UTR/nsp1 [9]) and MAYVx2-CHIKV duplex assay.
